# Altered functional activity in bipolar disorder: A comprehensive review from a large‐scale network perspective

**DOI:** 10.1002/brb3.1953

**Published:** 2020-11-18

**Authors:** Sujung Yoon, Tammy D. Kim, Jungyoon Kim, In Kyoon Lyoo

**Affiliations:** ^1^ Ewha Brain Institute Ewha W. University Seoul South Korea; ^2^ Department of Brain and Cognitive Sciences Ewha W. University Seoul South Korea; ^3^ Graduate School of Pharmaceutical Sciences Ewha W. University Seoul South Korea; ^4^ The Brain Institute and Department of Psychiatry University of Utah Salt Lake City UT USA

**Keywords:** emotion, executive control, functional magnetic resonance imaging (fMRI), psychiatric disorders

## Abstract

**Background:**

Growing literature continues to identify brain regions that are functionally altered in bipolar disorder. However, precise functional network correlates of bipolar disorder have yet to be determined due to inconsistent results. The overview of neurological alterations from a large‐scale network perspective may provide more comprehensive results and elucidate the neuropathology of bipolar disorder. Here, we critically review recent neuroimaging research on bipolar disorder using a network‐based approach.

**Methods:**

A systematic search was conducted on studies published from 2009 through 2019 in PubMed and Google Scholar. Articles that utilized functional magnetic resonance imaging technique to examine altered functional activity of major regions belonging to a large‐scale brain network in bipolar disorder were selected.

**Results:**

A total of 49 studies were reviewed. Within‐network hypoconnectivity was reported in bipolar disorder at rest among the default mode, salience, and central executive networks. In contrast, when performing a cognitive task, hyperconnectivity among the central executive network was found. Internetwork functional connectivity in the brain of bipolar disorder was greater between the salience and default mode networks, while reduced between the salience and central executive networks at rest, compared to control.

**Conclusion:**

This systematic review suggests disruption in the functional activity of large‐scale brain networks at rest as well as during a task stimuli in bipolar disorder. Disrupted intra‐ and internetwork functional connectivity that are also associated with clinical symptoms suggest altered functional connectivity of and between large‐scale networks plays an important role in the pathophysiology of bipolar disorder.

## Graphical abstract

The investigation of the brain alterations with a large‐scale network perspective may provide a more comprehensive way in elucidating the neuropathology of a disorder. This systematic review evaluates recent literature and suggests alteration in the functional activity of large‐scale brain networks including the default mode, salience, and central executive networks in bipolar disorder. Furthermore, disrupted intra‐ and internetwork functional connectivity are also associated with clinical symptoms of bipolar disorder, which highlight the importance of large‐scale networks in the pathophysiology of bipolar disorder.

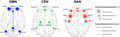

## 1 INTRODUCTION

Bipolar disorder is a neuropsychiatric disorder that is characterized by severe disturbance and fluctuation in mood (Rowland & Marwaha, 2018). According to the latest edition of the Diagnostic and Statistical Manual of Mental Disorders (DSM‐5) (American Psychiatric Association, 2013), bipolar and related disorders entail affective episodes of mania and depression as primary clinical symptoms (Severus & Bauer, 2013) along with symptoms of cognitive deficit (Douglas et al., 2018), together which may lead to an overall reduced quality of life (Saarni et al., 2010). Considering the high lifetime prevalence of bipolar spectrum disorder of 2.4% across the globe (Mathers et al., 2003; Merikangas et al., 2011), elucidating the pathophysiology of bipolar disorder is an urgent matter among other psychiatric illnesses (Ferrari et al., 2016).

Over the years, many studies investigated the structural changes of the brain in bipolar disorder through various neuroimaging approaches including voxel‐based morphometry (Lyoo et al., 2004; Selvaraj et al., 2012), tissue volumetric analysis (Vita et al., 2009), cortical thickness analysis (Hanford et al., 2016; Lyoo et al., 2006), and white matter alterations (Marlinge et al., 2014). Furthermore, recent literature has also taken functional magnetic resonance imaging (fMRI) data to identify specific brain regions that undergo significant alteration in functional activation patterns in bipolar disorder (Chen et al., 2011; Marchand & Yurgelun‐Todd, 2010). Collective findings from these studies may suggest the potential roles of certain cortical and subcortical regions of the brain as neural correlates of bipolar disorder. However, the associations between the multiple brain regions identified to be significantly altered as well as the clinical symptoms of bipolar disorder remain rather inconclusive. A recent review on the classification and prediction of brain disorders using functional connectivity discusses the advantages to the investigation of brain connectivity according to regions that are functionally co‐activated (Du et al., 2018). This is in alignment with other previous literature which notes the importance of identifying the functional connectivity in a multitude of brain regions that are known to have functional connections with a key anatomical brain region involved in the disorder (Pannekoek et al., 2014). Moreover, considering that a single brain region can belong to a number of overlapping brain networks (Xu et al., 2013) and its functional activation at a regional level may not fully explain for the functional alterations at a whole‐brain level, investigating the functional connectivity between multiple, anatomically remote yet functionally relevant brain regions may reveal more widespread information on the neural underpinnings of the disorder. In the case of bipolar disorder, a study also reported the significance of a particular brain region and its related functional connectivities as a significant marker that distinguishes bipolar disorder from healthy controls, particularly the connectivity between the basal ganglia to regions that make up the default mode network and the central executive network (Teng et al., 2013). Therefore, further investigation using a broader perspective, such as the observation of functional alterations from a network‐based approach, may expand the scope of our understanding on the neurological changes in bipolar disorder.

Recently, growing evidence suggest the significant roles of functional brain alterations in bipolar disorder, such as the functional connectivity within and between brain regions that are responsible for cognition and emotion (Cerullo et al., 2012; Han et al., 2019; Whalley et al., 2012). For instance, studies demonstrated that emotional instability, a primary symptom in bipolar disorder, is significantly associated with the functional connectivity among brain networks involving the limbic regions (Blond et al., 2012; Townsend and Altshuler, 2012). Moreover, a recent study suggests that the pattern of functional activation among particular brain regions may not only act as neural correlates of a key symptom in bipolar disorder, but also a potential biomarker that classifies bipolar disorder from other illnesses with similar clinical symptomatology such as major depressive disorder (Manelis et al., 2020) and schizophrenia spectrum disorders (Palaniyappan et al., 2019). This highlights the potential significance of exploring the functional connectivity of a particular network of brain regions as a reliable approach to further understanding bipolar disorder. In addition, considering that bipolar disorder includes a broad range of symptoms even within the domains of emotion and cognition such as affective instability, impulsivity, executive function, and memory (Phillips and Vieta, 2007)—all of which involve the activation of a number of brain regions—investigating brain regions associated with the symptoms of bipolar disorder from a network‐based perspective may provide a more comprehensive way to understanding the pathology of the disorder.

In recent years, large‐scale functional brain networks have become a growing interest in neuroimaging studies, as typically measured using signaling obtained through functional magnetic resonance imaging (fMRI) (Riedl et al., 2016). One of the key implications of recent network‐based studies is the identification of specific brain regions that are different in functional activity in bipolar disorder compared healthy controls. In addition to this, understanding the functional alterations of the brain at a network level that are characteristic of bipolar disorder may provide insight with regards to the major challenges in the current clinical field, such as difficulty in accurate disease diagnosis, delayed diagnosis due to inadequate characterization of symptoms, and the frequent comorbidity found in bipolar disorder (Angst & Cassano, 2005). Considering that distinct functional connectivity patterns at a network level have been identified in a depression model (Kaiser et al., 2015; Mulders et al., 2015), a similar approach may be taken to characterize bipolar disorder from a neurological perspective.

Among the many ways to categorize brain regions according to large‐scale networks, recent literature implicates a growing interest in the functional connectivity in intrinsic resting‐state networks (RSNs). Currently, functional connectivity of RSNs have been mostly explored to differentiate between bipolar disorder and other affective disorders such as unipolar depression (Han et al., 2019) or psychotic disorders like schizophrenia (Whalley et al., 2012). Also, since the recent review of the functional connectivity among intrinsic RSNs in bipolar disorder (Vargas et al., 2013), a growing number of original researches have been conducted, which may warrant for an updated review on this perspective. Findings on the relationship between intrinsic RSNs may improve the current limitations to bipolar disorder research, as it may help interpret the multiple brain regions that were previously suggested as individual neural correlates of bipolar disorder in a more comprehensive manner. The characterization of bipolar disorder acquired at a network level may then allow a detailed prognosis of the disorder, leading to an early onset of preventive measures, as well as the promotion of targeted treatment strategies to further explore treatment response. Furthermore, overseeing the multi‐regional alterations in functional connectivity in bipolar disorder may provide additional information regarding the neurological pathways that underlie bipolar disorder, which may then impart insight toward understanding the pathology of the disorder in a broader perspective.

The aim of this study was to review major findings from recent studies on bipolar disorder that investigated the brain functional networks of bipolar disorder. The review of a network‐based perspective may elucidate distinct intra‐ and internetwork connectivity alterations in bipolar disorder that can explain inconsistencies often found using more targeted approaches such as voxel‐based studies (Bora et al., 2010). For this review, we focused on original research articles that have been conducted within the past 10 years in a systematical assessment method. The study will summarize current findings on the network‐based alterations of bipolar disorder, as well as assess how these findings relate to recent findings on region‐based functional connectivity in bipolar disorder.

## 2 MATERIALS AND METHODS

### 2.1 Literature search strategy

A literature search was conducted on two electronic databases: PubMed and Google Scholar. The search was performed under a filter where studies published from January of 2009 through August of 2019 were included only. The following keywords were used as search terms: *bipolar disorder*, *affective disorder*, *mood disorder*, *brain imaging*, *magnetic resonance imaging*, *network*, and *functional network*. Specifically, an advanced search strategy was conducted with the above terms used either alone or in combination in the search. Following the literature search, the records identified were reviewed where duplicates were removed, and the titles and abstracts of the remaining records were assessed for eligibility as according to the following eligibility criteria.

### 2.2 Eligibility criteria

All studies included in the current review were eligible original research that satisfied the following criteria: (a) in English language, (b) primary target population of individuals with bipolar disorder, (c) brain functional magnetic resonance imaging data as one of the outcome variables, and (d) evaluates the functional connectivity or functional network of the brain. Any review papers, conference abstract, short communications were excluded.

### 2.3 Methodology of analysis for brain functional connectivity

For the current review, functional connectivity was defined as follows: (a) spatial or temporal alterations in blood oxygen level‐dependent (BOLD) signals (Lee et al., 2013), (b) measures of connectivity between two cortical or subcortical regions using path length, participation index, or clustering coefficient (Roberts et al., 2017), or (c) the amplitude of regional neuronal activity as measured by amplitude of low‐frequency fluctuations (ALFF) as previously described (Zou et al., 2008). Studies with designs that fell within these definitions were included as part of the literature review, including those that measured the functional connectivity using resting‐state BOLD signals, as well as those that have investigated task‐based BOLD signals. For analytical approaches to measuring functional connectivity, the current review includes some of the primary methodologies that are most common in recent literature. In particular, hypothesis‐driven approaches that are reviewed are seed‐based or region‐of‐interest (ROI) analysis and ALFF analysis. ROI analysis calculates the temporal correlation of a priori selected seeds with other regions of the brain, while ALFF measures the ratio of low‐frequency fluctuations to that of the whole frequency within a specific a priori brain region in order to estimate the regional neuronal activity level (Zou et al., 2008). The review also includes studies using the data‐driven approach of independent component analysis (ICA), which divides the whole brain into a number of independent components (ICs), resulting in the extraction of functional maps across time (Beckmann & Smith, 2004).

### 2.4 Ethical statement

We confirm that all authors have read the Journal's position on issues involved in ethical publication and affirm that this report is consistent with those guidelines.

## 3 RESULTS

### 3.1 Results from literature search

**FIGURE 1:**
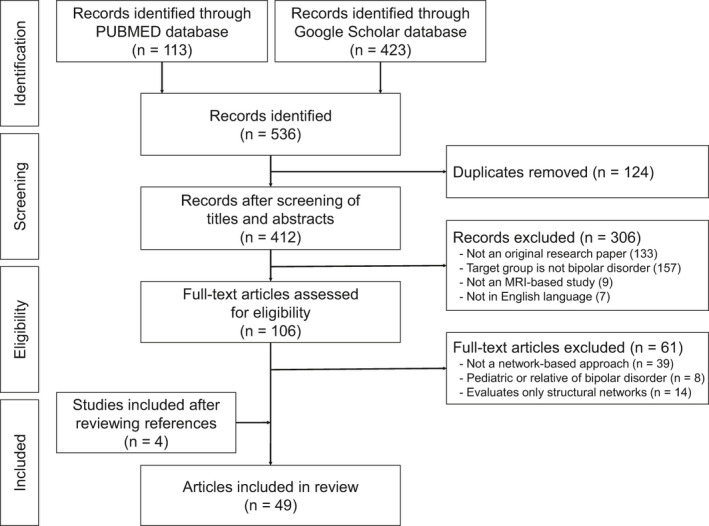
Flowchart of literature search and identification of eligible articles. The initial database search resulted in 113 records from PubMed and 423 records from Google Scholar, together which resulted in 412 records after the exclusion of duplicates. The first screening of titles and abstracts excluded 306 records for the following reasons: 133 records were not an original research paper, 157 records did not target bipolar disorder, 9 records did not include MRI data, and 7 records were not in English. A second screening of the 106 full‐text articles led to the exclusion of 61 records as a result of the following: 39 records did not use a network‐based approach, 8 records were based on pediatric or relatives of bipolar disorder, and 14 records evaluated structural brain networks. An additional four records were also included from cross‐referencing, resulting in a total of 49 records for this review. MRI, magnetic resonance imaging.

A total of 536 articles were identified after the literature search. First, duplicates were removed, followed by the screening of titles and abstracts of the studies. The screening process excluded 306 studies as according to the eligibility criteria mentioned above. A full‐text review was then conducted, where a total of 61 articles were excluded after careful consideration. Specifically, 39 articles were removed because the scope of the studies did not include a network‐based approach, 8 articles were excluded due to having a sample population of either a pediatric bipolar disorder patients or healthy relatives of bipolar disorder patients, and 14 articles were removed as they only evaluated the structural networks of the brain. A total of 49 studies were identified and included in this review, including 4 studies that were obtained through cross‐reference. Of these, 30 were ROI‐based studies that investigated the functional connectivity among two or more regions of the brain, 16 were ICA‐based studies that investigated the alteration in functional connectivity within and between functional brain networks, and 3 were ALFF‐based studies which observed the activity in slow fluctuations between brain regions that are representative of specific RSNs. A detailed summary of the literature search process in identifying original articles eligible for the current review is shown in Figure 1.

Collectively, the 49 studies consisted of 1,782 individuals with bipolar disorder and 2,310 healthy controls. While approximately half of the studies specified the type of bipolar disorder of the participants enrolled, information with regard to the episode in bipolar disorder such as whether participants were in the state of mania, depression, or euthymia at the time of the assessment were often missed. In addition, medication status of the participants also varied across the 49 studies, where 11 studies consisted strictly of unmedicated bipolar disorder participants, 13 studies had both unmedicated and medicated bipolar disorder participants, 20 studies looked at bipolar disorder participants under various medications, and five studies did not provide relevant data. Furthermore, most of the study designs had measures of brain functional connectivity as the primary outcome variable, with some studies providing further exploratory analyses as to provide clinical implications to the functional network alterations in bipolar disorder. The studies also varied in terms of neuroimaging techniques including the scanner platform used, field strength, and MRI acquisition parameters. A summary of the neuroimaging techniques of the 49 studies reviewed is provided in Table S1.

### 3.2 Core functional networks altered in bipolar disorder

Of the 49 studies reviewed, 40 studies acquired resting‐state fMRI data, and therefore primarily explored the functional activity of brain regions that make up the major resting‐state networks. Brain regions that make up the default mode network (DMN), central executive network (CEN), and salience network (SAN) were most frequently used as a priori seeds in ROI‐based studies, and the same networks were also shown to undergo significant alteration in the majority of the ICA studies. Others that underwent significant alteration in functional connectivity included brain structures that make up the limbic network (LIM) and the cerebellar network (CBL).

The remaining 9 studies explored brain functional connectivity in bipolar disorder through task‐based fMRI. Research on task‐based fMRI focused on exploring the affective and cognitive symptoms of bipolar disorder through the selection of ROIs of the frontal cortex, specifically regions that make up the CEN. The nature of the tasks in these studies primarily included cognitive tasks such as those targeting working memory or affective tasks of negative and positive emotional stimuli.

Among the literature reviewed, studies that report altered brain functional connectivity in bipolar disorder using detailed anatomical brain regions as according to the Montreal Neurological Institute (MNI) template and cluster size are summarized in Table S2.

### 3.3 Network alteration according to ROI‐based studies

For studies that used the hypothesis‐driven approach of ROI analyses, functional alteration of large‐scale brain networks were assessed based on the networks to which the a priori ROIs belong, as well as the brain regions that demonstrated significant temporal correlations with the selected seeds as assessed through fMRI data. A total of 30 studies underwent seed‐based ROI analyses as to investigate the altered functional connectivity in bipolar disorder, one of which explored the functional connectivity of networks using a graph theory‐based approach (Dvorak et al., 2019). Of these, 18 of the studies had seed regions that make up the default mode network (DMN), 15 consisted of seed regions that make up the central executive network (CEN) (also termed as the frontoparietal control network or the executive control network in certain studies), and 14 consisted of seed regions that make up the salience network (SAN) (also coined with the cingulo‐opercular network due to consisting of similar seeds). A detailed summary of the ROI‐based studies reviewed is provided in Table 1.

**TABLE 1:** Summary of the large‐scale functional brain networks altered in bipolar disorder according to ROI‐based studies

Study (1st author)	Imaging modality	BD type	*N* (BD/HC)	Functional network	Altered direction	Clinical implications	Seeds of significant functional connectivity
Anand et al. (2009)	rs‐fMRI	BD	11/15	SAN‐LIM	Decrease	Greater severity of mood dysregulation	AMYG, DMTHAL, perigenual ACC, pallidostriatum
Anticevic et al. (2013)	rs‐fMRI	BD I	68/51	DMN, CEN‐LIM	Decrease, Increase	Greater severity of lifetime psychotic symptoms	AMYG, DLPFC, mPFC
Baker et al. (2014)	rs‐fMRI	BD	40/100	CEN	Decrease	Disrupted ability to distinguish between internal and external oriented processing	DLPFC, lateral parietal cortex, postero mPFC, posterior temporal cortex
Chai et al. (2011)	rs‐fMRI	BD	14/15	CEN‐DMN, SAN‐DMN	Decrease, Increase	Overly heightened attention that leads to disturbed responses to salient stimuli	Anterior INS, DLPFC, mPFC, VLPFC
Chen et al. (2019)	rs‐fMRI	BD II	90/100	DMN‐CBL	Decrease	Neurobiological feature of BD II depressed state	Crus I, Crus II, MFG, mPFC, precuneus
Dell'Osso et al. (2015)	task‐fMRI	BD I, II	28/27	CEN	Increase	Brain functional abnormality occurs prior to emergence of cognitive deficit in BD	DLPFC
Dvorak et al. (2019)^a^	rs‐fMRI	BDE	20/30	LIM	Decrease	Distinct emotional information processing than individuals with MDD	Caudate, fusiform gyri, hippocampus, MFG, MTG, olfactory cortex, putamen
Ellard et al. (2018)	rs‐fMRI	BD	24/39	SAN‐CEN, SAN	Decrease	Greater impairment in perceived emotion and reward sensitivity	INS, IPL
Ellard et al. (2019)	task‐fMRI	BD I	39/36	DMN, CEN, SAN‐CEN	Decrease, Increase	Impaired performance accuracy in cognitive task	Anterior INS, DLPFC, DMPFC, IPL, VLPFC
Favre et al. (2014)	rs‐fMRI	BDE	20/20	CEN‐DMN DMN‐LIM	Decrease, Increase	Longer disease duration	AMYG, DLPFC, mPFC
Gong et al. (2019)	rs‐fMRI	BD II	96/100	DMN, SAN	Decrease	Underlies depressive state of BD II	ITG, mPFC, PCC, precuneus, subgenual ACC
He et al. (2018)	rs‐fMRI	BD I, II	32/43	DMN‐CBL, CEN‐CBL	Decrease	Disrupted self‐referential and affective processing	Cerebellar vermis IV/V, cerebellar lobule IX, Crus I, DLPFC, precuneus, subgenual ACC
He et al. (2019)	rs‐fMRI	BD	25/34	CEN‐LIM	Increase	Longer average duration per episode	DLPFC, precuneus, striatum
Karcher et al. (2019)	rs‐fMRI	BD	40/60	SAN	Decrease	Impaired information integration	–
Li, Tang, et al. (2018)	rs‐fMRI	BD	46/66	SAN‐DMN, SAN‐LIM	Increase	Underlies shared cognitive and emotional dysfunction as in schizophrenia	Anterior INS, dorsal INS, fontal pole, MFG, perigenual ACC, THAL
Liu et al. (2019)	rs‐fMRI	BD	23/24	DMN, DMN‐LIM	Decrease	Greater severity of depression (HDRS)	AMYG, hippocampus, inferior occipital gyrus, MTG, PCC, precuneus, THAL, SFG, STG
Luo et al. (2018)	rs‐fMRI	BD II	94/100	DMN‐CBL, CEN‐CBL	Decrease	Disruption in cognition and self‐referential mentality	ACC, crus Ia/Ib, DLPFC, mPFC, MTG, ITG
Magioncalda et al. (2015)	rs‐fMRI	BD	40/40	DMN, SAN	Decrease	Greater severity of depression (HDRS), mania (YMRS), and cognitive deficit	ITG, PCC, supragenual ACC
Mamah et al. (2013)	rs‐fMRI	BD	35/33	SAN, SAN‐CBL	Decrease	Greater disorganization symptoms	–
Marchand et al. (2014)	task‐fMRI	BD II	19/18	DMN	Increase	Neurobiological trait pathology of BD	Dorsolateral SFG, medial SFG, SFG
Oertel‐Knöchel et al. (2015)	rs‐fMRI	BD	21/21	SAN‐DMN	Increase	Impaired episodic memory	MFG, MTG, SFG, STG, ACC
Pang et al. (2018)	rs‐fMRI	BD	30/30	SAN, SAN‐DMN	Decrease	Greater severity of depression (HDRS)	Dorsal anterior INS, ventral anterior INS, VLPFC
Pompei et al. (2011)	task‐fMRI	BDE	39/48	SAN‐CEN	Decrease	Dysfunctional integration of networks	DLPFC, INS, ventral ACC, VLPFC
Radaelli et al. (2015)	task‐fMRI	BD I	52/40	CEN‐LIM	Decrease	Abnormal modulation of emotion	ACC, AMYG, DLPFC
Rey et al. (2016)	rs‐fMRI	BD, BDE	27/27	SAN‐DMN, DMN‐LIM	Increase	Disrupted mood, increased rumination	AMYG, PCC, subgenual ACC, VLPFC
Roberts et al. (2017)^a^	rs‐fMRI	BD I, II	49/80	CEN‐DMN	Decrease	Intrusive emotional rumination	ITG, putamen, STG, VLPFC
Rodríguez‐Cano et al. (2017)	task‐fMRI	BD I	26/26	DMN, CEN, CBL	Increase, Decrease	Dysregulation in response to sensory and emotional stimuli	Cerebellum, DLPFC, DMPFC, mPFC, precuneus,
Sheffield et al. (2017)	rs‐fMRI	BD	129/201	SAN	Decrease	Disrupted integration of information	–
Townsend et al. (2013)	task‐fMRI	BD I	30/26	SAN‐DMN, CEN‐LIM	Decrease	Disrupted ability to relapse into acute mood state	ACC, DLPFC, MFG, PCC, VLPFC
Wang, Zhong, et al., 2018	rs‐fMRI	BD II	25/25	DMN‐CBL	Decrease	Pathology of the state of remission in BD	Cerebellar lobule VIIb/VIIIa, hippocampus, IPL, PCC, paracentral lobule, precuneus, putamen, STG

Note

The altered functional connectivity of large‐scale functional brain networks in bipolar disorder as reported in the ROI‐based studies reviewed. The name of the large‐scale functional brain network was determined either by the authors themselves within the study and/or based on the major brain regions that were used as a priori seeds. The direction of alteration for each study is in reference to the respective healthy control group. Functional connectivity between brain regions were reported under the term “decrease” for both positive and negative connectivities, including cases where the polarity of the functional connectivity was reversed.

Abbreviations: ACC, anterior cingulate cortex; AMYG, amygdala; BD, bipolar disorder (type unspecified); BD I, bipolar 1 disorder; BD II, bipolar 2 disorder; BDE, euthymic bipolar disorder; CBL, cerebellar network; CEN, central executive network; DLPFC, dorsolateral prefrontal cortex; DMN, default mode network; DMPFC, dorsomedial prefrontal cortex; DMTHAL, dorsomedial thalamus; EMN, episodic memory network; HC, healthy control; HDRS, Hamilton Depression Rating Scale; INS, insula; IPL, inferior parietal lobule; ITG, inferior temporal gyrus; LIM, limbic network; MDD, major depressive disorder; MFG, middle frontal gyrus; mPFC, medial prefrontal cortex; MTG, middle temporal gyrus; *N*, sample size; PCC, posterior cingulate cortex; PFC, prefrontal cortex; ROI, region‐of‐interest; rs‐fMRI, resting‐state functional magnetic resonance imaging; SAN, salience network; SFG, superior frontal gyrus; STG, superior temporal gyrus; task‐fMRI, task‐based functional magnetic resonance imaging; THAL, thalamus; VLPFC, ventrolateral prefrontal cortex; VMPFC, ventromedial PFC; YMRS, Young Mania Rating Scale.

^a^ Network‐based statistics studies that compared the between‐group differences in functional connectivity strength of an a priori region with other regional connections and networks.

#### 3.3.1 Intranetwork functional connectivity

**FIGURE 2:**
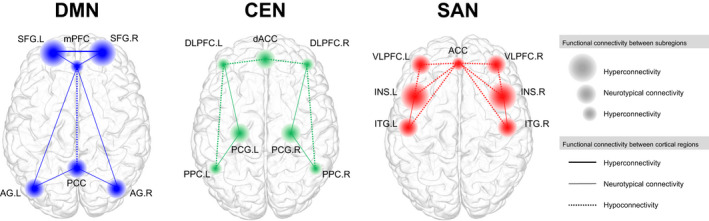
Intranetwork alteration in functional activity at rest based on current articles reviewed. Within‐network alteration in functional connectivity in bipolar disorder based on the triple network model. For each network, the key regions that belong to the network are shown in colored nodes. The size of the nodes represents hyperconnectivity, hypoconnectivity, or statistically similar (neurotypical) functional connectivity between the respective region and its nearby subregional structures, such as the functional connectivity between the dorsal and ventral part of the region, as compared to the healthy control group. The functional connectivity between regions are shown in edges, where the type of edge represents either hyperconnectivity, hypoconnectivity or statistically similar (neurotypical) functional connectivity as compared to the healthy control group. ACC, anterior cingulate cortex; AG, angular gyrus; CEN, central executive network; dACC, dorsal anterior cingulate cortex; DLPFC, dorsolateral prefrontal cortex; DMN, default mode network; INS, insula; ITG, inferior temporal gyrus; L, left; mPFC, medial prefrontal cortex; PCC, posterior cingulate cortex; PCG, precentral gyrus; PPC, posterior parietal cortex; SAN, salience network; SFG, superior frontal gyrus; R, right; VLPFC, ventrolateral prefrontal cortex

For ROI‐based studies, findings indicated reduced intranetwork functional connectivity at rest for networks belonging to the 3 major large‐scale RSNs, which are the DMN (Anticevic et al., 2013; Ellard et al., 2019; Gong et al., 2019; Liu et al., 2019; Magioncalda et al., 2015), CEN (Baker et al., 2014), and SAN (Gong et al., 2019; Karcher et al., 2019; Magioncalda et al., 2015; Mamah et al., 2013; Pang et al., 2018; Sheffield et al., 2017). In addition, the intranetwork functional connectivity of the LIM (Dvorak et al., 2019) was also decreased as compared to the respective control group. Interestingly, in the case of the CEN and DMN, the functional connectivity within the network during a task‐based stimuli such as emotion recognition or cognitive tasks were opposite in polarity with respect to at rest, with increased intranetwork functional connectivity of the CEN (Dell'Osso et al., 2015; Ellard et al., 2019) and DMN (Marchand et al., 2014) in individuals with bipolar disorder compared to their respective healthy control groups. A visual representation of the intranetwork functional connectivity alterations of the 3 major large‐scale RSNs at rest for individuals with bipolar disorder, compared to the respective healthy control group, is shown in Figure 2.

Among the large‐scale networks, the functional alteration of regions that make up the DMN were the most prevalent across studies. Specifically, some of the key regions that demonstrated significantly altered functional connectivity were the medial prefrontal cortex (mPFC) (Chai et al., 2011; Anticevic et al., 2013; Favre et al., 2014; Rey et al., 2016; Rodríguez‐Cano et al., 2017; Chen et al., 2019; Gong et al., 2019; Luo et al., 2018), posterior cingulate cortex (PCC) (Townsend et al., 2013; Magioncalda et al., 2015; Rey et al., 2016; Wang, Zhong, et al., 2018; Ellard et al., 2019; Gong et al., 2019), precuneus (Pang et al., 2018; Wang, Zhong, et al., 2018; He et al., 2019; Liu et al., 2019), and superior frontal gyrus (SFG) (Marchand et al., 2014; Rodríguez‐Cano et al., 2017; Karcher et al., 2019). Studies that selected the mPFC as one of the ROIs found reduced global brain connectivity of the mPFC with respect to the rest of the frontal cortex (Anticevic et al., 2013), as well as reduced functional connectivity between the mPFC and other regions of the DMN such as the PCC (Gong et al., 2019). For the SFG, findings have shown increased functional connectivity among subregions of the SFG also, where hyperconnectivity was found between the medial aspect of the left SFG and the dorsolateral region of the left SFG in bipolar disorder (Marchand et al., 2014).

For the CEN, an overall decreased functional connectivity between regions of the CEN (also described as the frontoparietal network in some studies) was reported (Baker et al., 2014; Mamah et al., 2013). Specific regions that demonstrated decreased functional connectivity were the connection between the dorsolateral prefrontal cortex (DLPFC) and the intraparietal sulcus (Mamah et al., 2013) as well as the lateral parietal cortex (Baker et al., 2014). Similar to the DMN, when performing a task, the functional connectivity of the CEN was increased as compared to the respective healthy control group (Dell'Osso et al., 2015; Ellard et al., 2019), except for the finding of one particular study which showed deactivation within the DLPFC (Rodríguez‐Cano et al., 2017).

Intranetwork functional connectivity of the SAN was also found to decrease at rest (Gong et al., 2019; Karcher et al., 2019; Magioncalda et al., 2015; Pang et al., 2018). Among the regions that make up the SAN, the insular cortex was the most involved seed across the studies reviewed (Townsend et al., 2013; Ellard et al., 2018; Li, Tang, et al., 2018; Pang et al., 2018; Ellard et al., 2019), followed by the putamen (Dvorak et al., 2019; He et al., 2019; Karcher et al., 2019), temporal gyrus (Gong et al., 2019), and perigenual anterior cingulate cortex (ACC) (Magioncalda et al., 2015). Specifically, functional connectivity was decreased between the anterior insula and ventrolateral prefrontal cortex (VLPFC) (Ellard et al., 2018; Pang et al., 2018), between the subgenual ACC and the inferior temporal gyrus (ITG) (Gong et al., 2019; Magioncalda et al., 2015) as well as between the perigenual ACC to the VLPFC (Magioncalda et al., 2015). Functional connectivity among the subregions of the ACC was reduced also, where reduced functional connectivity between the perigenual ACC to the supragenual ACC was reported (Magioncalda et al., 2015). In addition, reduced intranetwork functional connectivity of the cingulo‐opercular network (CON), which consists of brain regions that also make up key regions of the SAN, was reported in one study (Mamah et al., 2013). Furthermore, reduced global and local efficiency of the CON was found in one study, which was also significantly associated with cognitive ability (Sheffield et al., 2017).

For ROIs that make up large‐scale networks outside of the 3 major RSNs, individuals with bipolar disorder showed significantly lower nodal path length in the hippocampus, caudate, and fusiform gyri, which partially make up the LIM (Dvorak et al., 2019).

#### 3.3.2 Internetwork functional connectivity

**FIGURE 3:**
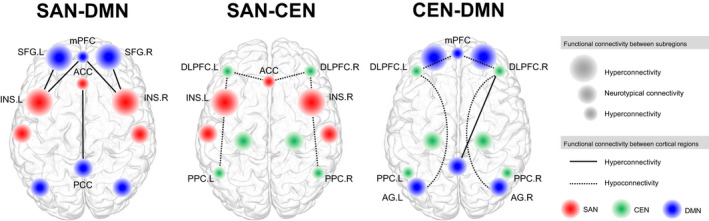
Internetwork alteration in functional connectivity at rest based on current articles reviewed. Internetwork alteration in functional connectivity in bipolar disorder based on the triple network model. Red represents major regions of the salience network, green represents major regions of the central executive network, and blue represents major regions of the default mode network. The size of the nodes represents hyperconnectivity, hypoconnectivity, or statistically similar (neurotypical) functional connectivity between the respective region and its nearby subregional structures, such as the functional connectivity between the dorsal and ventral part of the region, as compared to the healthy control group. In summary, the results from the 49 studies reviewed indicates a significant increased connectivity between the salience network and default mode network. Decreased functional connectivity was reported between the salience network and central executive network, as well as between the central executive network and the default mode network. It is noteworthy that increased functional connectivity was also found between the right dorsolateral prefrontal cortex of the central executive network and the posterior cingulate cortex of the default mode network, although this relationship was not reported bilaterally. ACC, anterior cingulate cortex; Angular gyrus; CEN, central executive network; DLPFC, dorsolateral prefrontal cortex; DMN, default mode network; INS, insula; L, left; mPFC, medial prefrontal cortex; PCC, posterior cingulate cortex; PPC, posterior parietal cortex; R, right; SAN, salience network; SFG, superior frontal gyrus

The collective findings from the ROI‐based studies demonstrated distinct internetwork functional connectivity patterns among large‐scale functional networks in bipolar disorder compared to healthy control. A visual representation of the internetwork functional connectivity alterations of the 3 major large‐scale RSNs at rest for individuals with bipolar disorder, compared to the respective healthy control group, is shown in Figure 3. The first distinct internetwork functional alteration is the hypoconnectivity of the CEN‐DMN in bipolar disorder as compared to control at rest. Two studies reported significant functional decoupling between the DLPFC of the CEN and mPFC of the DMN (Chai et al., 2011; Favre et al., 2014). In addition, one study which used network‐based statistics (NBS) confirmed the dysconnectivity between the frontal regions of the brain and the default mode network (Roberts et al., 2017). Interestingly, the alteration of functional connectivity between the CEN‐DMN was reversed in polarity when performing a working memory task, where the engagement between the DLPFC and the frontal gyrus was greater in individuals with bipolar disorder as compared to the healthy control group (Dell'Osso et al., 2015). For studies exploring the functional connectivity between regions that make up the CEN and DMN, studies reported an anticorrelation relationship between the two networks in the healthy control group, as described in the triple network model (Menon, 2011), while individuals with bipolar disorder demonstrated weakened anticorrelation as compared to the controls (Chai et al., 2011; Ellard et al., 2018; Favre et al., 2014). Among these, three reported that the anticorrelated relationship of CEN‐DMN weakened in the case of individuals with bipolar disorder as compared to healthy control (Chai et al., 2011; Favre et al., 2014; Roberts et al., 2017), while two studies reported findings that were quite the opposite (Marchand et al., 2014; Townsend et al., 2013).

Second, hyperconnectivity of the SAN‐DMN was reported. Specifically, increased functional connectivity was found between the insula of the SAN and mPFC of the DMN (Chai et al., 2011), and between the anterior cingulate cortex of the SAN and superior frontal gyrus of the DMN (Oertel‐Knöchel et al., 2015). Furthermore, the functional connectivity from the anterior insula cortex toward the middle frontal gyrus was demonstrated to be increased at rest (Li, Tang, et al., 2018), as well as between the ventral anterior insula and precuneus (Chai et al., 2011), and between the subgenual ACC and PCC (Rey et al., 2016). Similar to the internetwork functional relationship between the CEN‐DMN, the direction of alteration in functional connectivity of the SAN‐DMN reversed when performing a task, where functional connectivity among the ACC, PCC, medial frontal gyrus, and VLPFC (as well as the DLPFC of the CEN) decreased during emotion down‐regulation (Townsend et al., 2013).

Third, significantly altered functional connectivity of the SAN‐CEN was found (Pompei et al., 2011), where the polarity of the functional connectivity between the insula of the SAN and inferior parietal lobule (IPL) of the CEN was reversed in an anticorrelated manner (Ellard et al., 2018). In addition, this reduction was significantly associated with greater impairment in perceived emotion control, as well as impaired inhibition (Ellard et al., 2018), which are characteristics of mania (Christodoulou et al., 2006). In contrast, contradictory findings of increased functional connectivity between the dorsal anterior insula of the SAN and inferior IPL of the CEN was also reported at rest by one study (Pang et al., 2018). Interestingly, the increased functional connectivity between the SAN‐CEN was positively correlated with scores of the Hamilton Depression Rating Scale (HDRS) (Pang et al., 2018), which are often used as a measure of depression in bipolar disorder. During a cognitive task‐based stimuli, greater functional connectivity was reported between the SAN‐CEN in individuals with bipolar disorder compared to the control group, specifically among the anterior insula and DLPFC (Ellard et al., 2019).

Furthermore, large‐scale networks that were strengthened among each other which are the SAN and DMN each demonstrated increased functional connectivity with the LIM also (Favre et al., 2014; Rey et al., 2016; Li, Tang, et al., 2018), while the connectivity between the CEN‐LIM was decreased (Radaelli et al., 2015). However, one study reported opposite findings where the functional connectivity between the SAN‐LIM was decreased in bipolar disorder compared to control (Anand et al., 2009). In addition, significant alteration in functional connectivity between the mPFC of the DMN and amygdalae was reported in two studies, but the direction of alteration were inconsistent (Anticevic et al., 2013; Favre et al., 2014). The functional relationship between the task‐negative network, DMN, and the CBL, which is associated with cognition, was also explored in many studies. An overall reduced functional connectivity between the DMN‐CBL was reported in individuals with bipolar disorder compared to the respective healthy control group (He et al., 2018; Luo et al., 2018; Wang, Zhong, et al., 2018; Chen et al., 2019). In particular, reduced functional connectivity was reported between the mPFC and various cerebellar regions (Chen et al., 2019; Luo et al., 2018). Decreased connectivity of the CEN‐CBL was also reported by one study (Luo et al., 2018).

### 3.4 Network alteration according to ICA‐based studies

A total of 16 studies of the selected literature used the data‐driven approach of ICA analysis as to investigate the functional alteration of large‐scale brain networks. Of these, 10 studies explored the DMN, seven explored the CEN, 4 explored the SAN, and 8 investigated other miscellaneous functional network including the dorsal attention network, CBL, LIM, medial visual network, and sensorimotor network. A detailed summary of the ICA‐based and ALFF‐based studies reviewed is provided in Table 2.

**TABLE 2:** Summary of the large‐scale functional brain networks altered in bipolar disorder according to ICA and ALFF‐based studies

Analysis method	Study (1st author)	Imaging modality	BD type	*N* (BD/HC)	Functional network	Altered direction	Clinical implications
ICA‐based	Brandt et al. (2014)	task‐fMRI	BD I, II	100/100	CEN, DAN	Increase	More cognitive resources warranted perform a cognitive task
	Calhoun et al. (2012)	task‐fMRI	BD	48/62	DMN	Unspecified	Biomarker to distinguish from HC
	Das et al. (2014)	rs‐fMRI	BDE	16/13	SAN‐DMN	Increase	Impaired interaction between salience detection and self‐referential processing
	He et al. (2016)	rs‐fMRI	BD I, II	13/33	DMN	Increase	Greater severity of depression (HDRS)
	He et al. (2018)	rs‐fMRI	BD I, II	32/43	DMN‐CBL, CEN‐CBL	Decrease	Dysfunction in emotion processing
	Jimenez et al. (2019)	rs‐fMRI	BD	46/48	Medial visual network	Decrease	Lower ability of emotion management
	Lois et al. (2014)	rs‐fMRI	BD I	30/35	CEN‐LIM	Increase	Impaired top‐down control, abnormally increased bottom‐up interference prior to apparent cognitive deficit
	Meda et al. (2012)	rs‐fMRI	BD	64/118	DMN‐FON	Decrease	Reflection of vulnerability of psychosis
	Meda et al. (2014)	rs‐fMRI	BD	300/324	DMN	Decrease	Poorer social functioning and greater severity of clinical symptomatology
	Öngür et al. (2010)	rs‐fMRI	BD	17/15	DMN	Decrease	Greater severity of mania
	Skåtun et al. (2018)	rs‐fMRI	BD I, II	57/280	SMN	Increase	Symptoms of perceptual and cognitive distortions in BD
	Thomas et al. (2019)	rs‐fMRI	BD, BD I	42/52	CEN‐LIM (BD I), CEN‐SMN (BD)	Increase	Disturbance in regulation of attention, memory, decision‐making
	Wang, Wang, et al., 2018	rs‐fMRI	BD	38/47	SAN, CEN, DMN, DMN‐CEN, SAN‐DMN, SAN‐CEN	Decrease, Decrease, Increase	Disrupted self‐referential processing, processing of emotional information and emotional control
	Wang et al. (2019a)	rs‐fMRI	BD II	51/50	SAN, SAN‐DMN, CEN‐DMN	Decrease, Increase	Greater depression relapse
	Wang et al. (2019b)	rs‐fMRI	BD II	51/52	CEN‐DMN	Decrease	Dysfunctional information processing
	Yip et al. (2014)	rs‐fMRI	BD II	15/20	SAN‐LIM	Increase	Disrupted reward processing
ALFF‐based	Li, Liu, et al. (2018)	rs‐fMRI	BDE	21/28	LIM	Increase	–
	Liu et al. (2012)	rs‐fMRI	BD	26/26	CEN‐LIM	Unspecified	Greater severity of depression (HDRS)
	Wang et al. (2019)	rs‐fMRI	BD	30/31	CEN‐DMN	Unspecified	Greater cognitive imbalance

Note

The altered functional connectivity of large‐scale functional brain networks in bipolar disorder as reported in ICA‐based studies, as well as ALFF‐based studies. For ICA‐based studies, the altered direction refers to the alteration in overall functional connectivity within a large‐scale functional network or between multiple large‐scale functional networks. For ALFF‐based studies, the direction of alteration refers to the increase or decrease of frequency fluctuation, and does not necessarily indicate the strength of the functional connectivity. Unspecified altered direction indicates an alteration in functional connectivity that is statistically significant as compared to the respective healthy control group, without the provision of whether the alteration is increased or decreased.

Abbreviations: ALFF, amplitude of low‐frequency fluctuation; BD, bipolar disorder (type unspecified); BD I, bipolar 1 disorder; BD II, bipolar 2 disorder; BDE, euthymic bipolar disorder; CBL, cerebellar network; CEN, central executive network; DAN, dorsal attention network; DMN, default mode network; FON, fronto‐occipital network; HC, healthy control; HDRS, Hamilton Depression Rating Scale; ICA, independent component analysis; LIM, limbic network; *N*, sample size; rs‐fMRI, resting‐state functional magnetic resonance imaging; SMN, somatomotor network; task‐fMRI, task‐based functional magnetic resonance imaging.

#### 3.4.1 Intranetwork functional connectivity

Five studies reported altered functional connectivity within the DMN. Specifically, Calhoun and colleagues (2012) performed a 75‐component group ICA, where ICs that make up the DMN were significantly altered and was associated with the task stimuli. In He and colleagues (2016), 48‐component ICA was performed to demonstrate that greater functional connectivity strength is present in the DMN, including ICs that are composed of the superior and medial frontal regions as well as the cuneus, in individuals with bipolar disorder. Increased DMN functional connectivity was also reported in Das and colleagues (2014) between the precuneus and the rest of the DMN in bipolar disorder, which was associated with emotional awareness. In contrast, Meda and colleagues (2014) used a para‐ICA approach targeting the DMN to report reduced global functional connectivity in regions of the DMN including the mPFC, PCC, and precuneus as consistent with the ROI‐based study findings, and further suggest selective nodes within the DMN that may be heritable through genetic associations. The reduced functional connectivity of the DMN at rest was also supported in studies (Öngür et al., 2010; Wang, Wang, et al., 2018).

Brandt and colleagues (2014) performed a 36‐component principal component analysis (PCA) to demonstrate that ICs that make up the CEN and the dorsal attention network are significantly altered in individuals with bipolar disorder compared to the healthy control at task‐based situations, although the direction of this alteration was not specified. In addition, weaker functional connectivity among the anterior insula of the SAN, and the DLPFC of the CEN was also reported (Wang, Wang, et al., 2018). Findings also showed that weaker intranetwork functional connectivity was prevalent for all three of the triple network model (Wang et al., 2019a), where more time was spent in the phase where all 3 networks have sparse functional connections as compared to the healthy control group.

Other large‐scale functional network that underwent intranetwork functional connectivity alterations in bipolar disorder included the increased functional connectivity of the sensorimotor network (Skåtun et al., 2018), which was associated with symptoms of perceptual and cognitive distortion in the case of individuals with bipolar disorder.

#### 3.4.2 Internetwork functional connectivity

Significant altered functional connectivity between the three large‐scale networks of the triple network model was most prevalent across ICA‐based studies. First, similar to findings from the ROI‐based analyses, increased functional coupling of the SAN‐DMN was found in bipolar disorder using a 20‐component ICA (Das et al., 2014). Lois and colleagues (2014) used a 40‐component group ICA method and demonstrated significant group differences in the functional connectivity between the SAN‐CEN, where the polarity of the functional connectivity was opposite in the bipolar disorder group in reference to the healthy control group. Similar findings were reported in terms of decreased functional connectivity of the CEN‐DMN (Wang et al., 2019b). However, another study reported stronger functional connectivity among the CEN‐DMN, specifically between the DLPFC of the CEN and angular gyrus of the DMN (Wang, Wang, et al., 2018). In addition, contrary to the ROI‐based findings, decreased functional connectivity of the SAN‐DMN at rest was reported also (Wang, Wang, et al., 2018). Here, it is noteworthy that the SAN and the meso/paralimbic network were identified as a single component, and the CEN was referred to as the frontoparietal network.

For the internetwork connectivity between networks of the triple network model and other large‐scale networks, He et al. (2018) showed decreased functional connectivity between independent components that make up the DMN (precuneus), CEN (DLPFC), and SAN (subgenual ACC), respectively, and the CBL, a finding which was performed using ROI‐based analysis then validated through an ICA approach. However, in contrast, Wang et al. (2019b) reported increased functional activity between the SAN‐CBL. In addition, the CEN functional activity was reported as more strongly connected with the caudate in individuals with bipolar disorder type 1, while those with bipolar disorder (type unspecified) showed greater functional activity of the sensorimotor network with the precentral gyrus of the CEN (Thomas et al., 2019). Furthermore, Meda et al. (2012) identified decreased functional connectivity between the anterior DMN and fronto‐occipital network as well as increased functional connectivity between the LIM and frontotemporal network, which in turn correlated with negative clinical symptoms as according to the Positive and Negative Symptom Scale (PANSS) (Meda et al., 2012). Yip et al. (2014) also reported significant functional connectivity alteration in bipolar disorder type 2 participants, where increased coherence across the insula of SAN and regions of the LIM including the putamen was reported at rest. Other networks with significant alteration included the significant alteration between thalamic regions, which make up the LIM and the somatomotor network (SMN) (Skåtun et al., 2018).

### 3.5 Network alteration according to ALFF‐based studies and others

Three studies using ALFF as outcome measures to investigate functional brain network alteration were selected for review. Of these, Li, Liu, et al. (2018) reported decreased functional connectivity within the LIM, specifically through higher ALFF levels in the amygdala and supplementary motor area in individuals with bipolar disorder compared to the healthy controls. Liu and colleagues (2012) reported that those with depressive bipolar disorder had significantly higher ALFF in the left insula of the SAN along with other temporal regions, as well as the CBL. In contrast, Liu et al. (2012) demonstrated lower ALFF in the parahippocampal regions in bipolar disorder. Lastly, Wang X and colleagues (2019) showed significantly greater fractional ALFF in the superior frontal gyrus of the DMN, a finding that also correlated with decreased gray matter volumes in the respective regions.

Apart from the triple network model, Jimenez et al. (2019) used nine key RSNs using the Smith atlas to generate subject‐specific maps and showed significant reduced functional connectivity in the medial visual network of bipolar disorder compared to control which also correlated with emotion management.

## 4 DISCUSSION

### 4.1 Summary of main findings

The aim of the current systematic review was to examine the current trends in existing literature on network‐based study findings in bipolar disorder. Through a systematic search of literature, a total of 49 studies were reviewed, of which the methodologies and key findings were explored. A majority of the studies reviewed demonstrated altered functional connectivity of functional brain networks through the hypothesis‐driven approach of a priori ROI analyses, where brain regions that are most representative of a specific large‐scale network were selected as seeds. Other methodologies exploring the functional connectivity of the brain in bipolar disorder included the ICA, ALFF, and network‐based statistics.

In general, in terms of intranetwork functional activity, patients with bipolar disorder generally show reduced functional connectivity within each of the triple network model which are major RSNs as according to previous literature (Menon, 2011). Reduced functional connectivity was reported between regions that make up the DMN (Öngür et al., 2010; Anticevic et al., 2013; Meda et al., 2014; Magioncalda et al., 2015; Wang, Wang, et al., 2018; Gong et al., 2019; Liu et al., 2019), CEN (Baker et al., 2014; Wang, Wang, et al., 2018), and SAN (Ellard et al., 2018; Gong et al., 2019; Karcher et al., 2019; Magioncalda et al., 2015; Mamah et al., 2013; Pang et al., 2018; Sheffield et al., 2017) at rest. Specific regions showed increased functional connectivity at a subregional level also, such as between the medial and dorsolateral parts of the SFG of the DMN (Marchand et al., 2014) and the dorsal and ventral regions of the anterior insular cortex of the SAN (Li, Tang, et al., 2018; Wang, Wang, et al., 2018). In particular, the altered within‐network functional connectivity of the intrinsic brain networks such as the DMN in bipolar disorder was associated with the symptoms of depression (MADRS) (He et al., 2016) and lifetime psychosis history (Anticevic et al., 2013). These findings are in alignment with previous literature, which emphasized the clinical significance of disrupted DMN in mood disorders (Mohan et al., 2016; Zhong et al., 2016), as well as in anxiety disorders (Coutinho et al., 2016; Lanius et al., 2010). Specifically, these studies noted that disruption in the DMN functional activity was associated with symptoms of depression, anxiety, or suggested as reliable predictors of related clinical symptom severity. In addition, the significant relationship between the functional activity of the DMN and clinical symptoms in mood disorders was also validated through the significant normalization of DMN function in depressed individuals after antidepressant treatment (Qin et al., 2015). Studies reviewed here support previous findings of disruption in particular large‐scale networks of the brain in bipolar disorder, and in particular, networks that make up the triple network model.

The studies reviewed in the current article also provide supportive evidence for previous voxel‐based neuroimaging studies that demonstrated significant structural and functional alteration in bipolar disorder. In particular, brain regions that indicated significant altered functional connectivity between its subregional structures were similar to regions that underwent significant gray matter alteration in previous literature. To name a few, significant gray matter volume reduction was reported in the mPFC (Liu et al., 2012), bilateral cingulate cortex (Lochhead et al., 2004), posterior parietal cortex and parietal lobule (Adler et al., 2005), and dorsolateral prefrontal cortex (Tost et al., 2010), as well as increased volume in the insula (Lochhead et al., 2004).

Among these, the functional connectivity of the mPFC was particularly of interest across the studies reviewed. The mPFC has been described to play a crucial role in self‐reflection (Passingham et al., 2010), and is associated with important cognitive and affective functions such as emotional facial recognition (Keener et al., 2012). The collective findings from the studies reviewed demonstrated reduced functional connectivity within the mPFC and increased functional connectivity between the mPFC and insula in individuals with bipolar disorder as compared to healthy controls. One of the ways to interpret this alteration may be based on a previous study, which describes increased functional connectivity as a functional compensatory response in bipolar disorder (Wessa et al., 2014). The hyperconnectivity between the mPFC of the DMN and insula of the SAN as reported in the current studies reviewed may indicate a compensation for the lack of mPFC connectivity with other regions in the case of bipolar disorder, and may enable adequate functioning of the brain at rest as well as salience detection.

Another interpretation to the reduced connectivity within the mPFC and hyperconnectivity of the SAN‐DMN may follow the paradigm of attentional shift as described in the triple network model (Menon, 2011). According to the triple network model, dynamic interactions are present between networks in order to regulate the shift between attention and access to cognitive resources, and this shift is supported by greater activity in the frontal region (Menon, 2011). Considering that reduced functional connectivity within the mPFC have been suggested to represent disruption in emotional processing (Etkin & Wager, 2007), the reduction in functional connectivity of the mPFC may be a neural characteristic of affective disorders including bipolar disorder. Furthermore, studies have also noted that increased activation of the insula may be a misattribution of emotional salience and lead to increased symptoms of anxiety, neuroticism, and in schizophrenia, psychosis (Menon & Uddin, 2010). Dysfunctional saliency processing has also been noted to lead to diminished cognitive capabilities (Menon, 2011). Therefore, the current findings may suggest reduced functional connectivity within the mPFC of the DMN and the increased activation of the insula as characteristics of disrupted emotional processing and severe mood in bipolar disorder.

From an internetwork perspective, the articles reviewed indicate a significant disruption across functional networks in bipolar disorder. Specifically, many of the studies demonstrated hyperconnectivity between the DMN and SAN at rest, while the relationship between the CEN‐SAN and CEN‐DMN indicated hypoconnectivity. Considering the anticorrelation nature of the CEN and DMN in normal healthy conditions (Menon, 2011), the weakened or reversed functional connectivity between the two networks as reported in the studies currently reviewed may indicate an imbalance in the brain's ability to segregate between the state of rest and a task‐positive state in the case of bipolar disorder. The increased functional activity involving the CEN during the performance of a cognitive or emotion‐related task may also indicate the increased need to compensate for the decreased ability to enable emotional and/or cognitive processing at rest in bipolar disorder (Rey et al., 2016) due to the ongoing disruption of the brain involving affective disorientation. Das et al. (2014) has also noted the increased functional coupling between the DMN‐SAN as a compromise of the weakened social salience detection and self‐referential processing in individuals with affective problems such as in bipolar disorder.

### 4.2 Limitations

A few limitations should be noted. First, the current review provided an overview of functional brain alterations from a network perspective focusing on the triple network model, along with some of the other major networks such as the limbic network and cerebellar network. Due to this, the current review has simplified the names of the large‐scale networks of interest according to regions that overlap, including the converging of the anterior and posterior DMN, the SAN and the cingulo‐opercular network, the CEN and the frontoparietal control network, and the LIM with the rest of the basal ganglia. While the triple network model has been a growing interest in many disorders including PTSD (Lanius et al., 2010), MDD (Kaiser et al., 2015; Mulders et al., 2015; Zhong et al., 2016), and schizophrenia (Meda et al., 2014; Öngür et al., 2010), future studies that investigate the precise findings in functional connectivity according to each large‐scale network may help elucidate a more detailed neurobiological pathway underlying bipolar disorder.

Second, it is noteworthy that the current search strategy led to the inclusion of two main approaches to the investigation of functional alteration of the brain which are the ROI analyses and ICA analyses. While the two methods of analyses represent both the hypothesis‐driven and data‐driven approaches to exploring the functional connectivity of the brain, it is noteworthy that very few articles using other methods such as NBS of ALFF were included. Through ROI‐based studies, precise alteration among a priori seeds were precisely reported, highlighting the advantage of exploring functional deficits in regions that are known to be associated with the disorder. In contrast, ICA‐based analyses allow an overall view of brain connectivity (Vargas et al., 2013), which enables a more exploratory approach to the investigation of neural correlates of bipolar disorder. However, other techniques that are noted as promising methods of research should also be noted and discussed in future reviews. For instance, researchers have described the applications of graph theory which enables the precise computation of networks according to nodes and edges (Peyron & Fauchon, 2019), or global brain connectivity, which examines the functional connectivity at a voxel level (Hahamy et al., 2014) as important techniques. Future reviews that explore various techniques and measures of functional alteration of the brain in bipolar disorder is warranted. In addition, it is also noteworthy ICA approaches allow the overlap between networks to account for the brain regions that belong to more than one network (Pletzer et al., 2016). The overlap of cortical structures as a result of the respective regions participating in a number of functions across networks has been noted previously (Haller & Bartsch, 2009), making it difficult to interpret the altered functional connectivity between regions at a network level and its clinical implications. For instance, the CEN is composed of the anterior insula, operculum, dorsal anterior cingulate cortex, and thalamus, all of which may overlap with a part of the LIM or SAN (Sadaghiani & D'Esposito, 2014). Therefore, further discussion is warranted in the development of a fixed guideline as to the large‐scale networks to which each seed belongs.

Third, the current systematic review did not include other methods to investigate altered brain functional connectivity such as regional homogeneity or expansion of ROI‐based approaches using various atlases for parcellation of seed selection. The addition of supportive evidence through other methods as mentioned above may provide more conclusive patterns of altered intra‐ and internetwork functional connectivity in bipolar disorder.

Fourth, it is noteworthy that 9 of the 49 studies currently reviewed investigated the functional connectivity of the brain in bipolar disorder using a task‐based fMRI study design. While this may be a small proportion compared to the majority of the studies reviewed which explored the neurological alterations of bipolar disorder through a resting‐state fMRI study design, research has shown that the two MRI acquisition techniques have very distinct functional characteristics (Friedman et al., 2006). The merging of findings for studies that explored the functional network of the brain at rest with those during a task‐based stimuli may challenge the current summary of results. Here, it is also noteworthy that, even within task‐based fMRI studies, additional factors such as the difficulty of the task have also been considered as important variables to consider (Manelis et al., 2020). Similarly, growing literature also demonstrates that dynamic functional connectivity provides more informative results than static functional connectivity, and notes the potential shortfalls of the underlying assumption in fMRI studies that functional connectivities of brain networks are stationary (Du et al., 2017; Zhi et al., 2018). Future research that narrows the scope of the review to one of the two fMRI techniques or provides a more extensive literature review including studies that explored the dynamic functional connectivity of large‐scale brain networks in bipolar disorder is warranted.

Lastly, the current review article does not discuss the key findings while considering other additional factors associated with bipolar disorder, such as the segregation of results according to the state and mood of bipolar disorder, as well as medication status of the participants. In particular, it is noteworthy that while the majority of the studies discussed in this review provided some sort of information regarding the medication status and history of the bipolar disorder participants, only 4 of the studies reported medication status in a detailed manner where medication load was calculated in the form of ordinal data or composite scoring. While the state and mood of bipolar disorder as well as medication status may be important factors to consider in developing a more extensive and comprehensive understanding of bipolar disorder from a neurological perspective, they have not been actively discussed in this review due to the limited number of studies that provided the relevant data. For instance, previous research reported significant differences in the functional networks altered according to the type of bipolar disorder (Thomas et al., 2019), while another discussed the potential limitations of interpreting findings on brain activity due to disparity in medication status or the complexity of the medication regimen (Chepenik et al., 2010). Therefore, future studies that also review the state and mood of bipolar disorder as well as the presence of concomitant pharmacotherapy may provide more consistent findings at a network level. Furthermore, the consideration of other important factors that contribute to the complexity of bipolar disorder including genetic polymorphisms (Meda et al., 2014; Oliveira et al., 2015), age of onset (Oliveira et al., 2015), and environmental factors such as maltreatment at an early age (Agnew‐Blais & Danese, 2016) is warranted.

## 5 CONCLUSIONS

In conclusion, the current review suggests the functional activity of the triple network model at rest and at task‐based stimuli as potential neural correlates for bipolar disorder as compared to healthy individuals. While the current literature review was construed under the assumption that functional connectivity between two brain regions imply functional interactions between two brain networks to which each brain region belongs, the present approach enables the identification of potential networks of interest in revealing the neural correlates of bipolar disorder. The collective findings of recent literature indicated that individuals with bipolar disorder have overall weakened function of the task‐negative state, salience detection, and cognitive control at rest, which may result to the dysfunction in alternating between task‐positive and task‐negative states and lead to difficulties in affective and cognitive function. Furthermore, such weakened activity across the triple network model may potentially lead to a compensatory response between the task‐negative and salience networks at rest, and hyperconnectivity of the cognitive network when performing a task. Studies that used the hypothesis‐driven approach of ROI analysis were most prevalent and easily interpretable in terms of the functional relationship among key brain regions, while the majority of the ICA‐based studies provided supportive evidence of the ROI findings through a data‐driven approach at a network level. The current study may demonstrate the significance of network‐based perspectives in bipolar disorder, as it may provide more comprehensive results on the alterations of the brain and lessen the likelihood of errors in selection of regions of interest. The elucidation of the particular large‐scale networks as potential neural correlates of bipolar disorder may then be used as potential strategies to reduce misdiagnosis of bipolar disorder, help distinguish bipolar disorder from other comorbid illnesses and those with similar clinical symptomatology, as well as understanding the diagnostic state of the disorder. Further research on functional MRI studies using a network‐based approach, including longitudinal studies and interventive studies, may help determine the directionality of the tentative internetwork pathways speculated in this review.

## CONFLICT OF INTEREST

None declared.

## AUTHOR CONTRIBUTION

IKL developed the original idea of the study and supervised the study. SY and TDK retrieved the data. SY, TDK, and JK abstracted and analyzed the data. All the authors wrote and approved the manuscript.

## Supporting information

App S1

## Data Availability

Data sharing is not applicable to this article as no new data were created or analyzed in this study.
