# Supplementary material for: Altered functional activity in bipolar disorder: A comprehensive review from a large‐scale network perspective
Source: Brain Behav. 2020 Nov 18;11(1):e01953. doi: 10.1002/brb3.1953 (PMC7821558; doi:10.1002/brb3.1953)
Supplement: Supplementary file 1 — App S1 [file BRB3-11-e01953-s001.docx]

**Supplementary Information for the manuscript:**

Altered functional activity in bipolar disorder: A comprehensive review from a large-scale network perspective

Sujung Yoon^1,2^, Tammy D. Kim^1^, Jungyoon Kim^1,2^, In Kyoon Lyoo^1,2,3,4^

^1^ Ewha Brain Institute, Ewha W. University, Seoul, South Korea

^2^ Department of Brain and Cognitive Sciences, Ewha W. University, Seoul, South Korea

^3^ Graduate School of Pharmaceutical Sciences, Ewha W. University, Seoul, South Korea

^4^ The Brain Institute and Department of Psychiatry, University of Utah, Salt Lake City, Utah, USA

**Correspondence**

In Kyoon Lyoo,

Ewha Brain Institute and Department of Brain and Cognitive Sciences, Ewha W. University, 52 Ewhayeodaegil, Seodaemungu, Seoul, 03760, South Korea.

Email: inkylyoo@ewha.ac.kr

**CONTENT**

Supplementary Table 12

Supplementary Table 26

| **Supplementary Table 1. Functional magnetic resonance imaging device information and acquisition parameters** | | | | | | | | | |
| --- | --- | --- | --- | --- | --- | --- | --- | --- | --- |
|  | | | | | | | | | |
| **Reference** | **Scanner** | **Field Strength** | **Eye State / Design of fMRI** | **fMRI Acquisition Parameters** | | | | | |
|  |  |  |  | **Volumes / Voxel Size** (mm) | **TR / TE**  (ms) | **Slice Thickness**  **/ Gap** (mm) | **FOV**  (mm) | **Matrix**  (mm) | **FA**  (°) |
| Anand et al. (2009) | GE | 1.5T | Close / Rest | 166 / - | 2,000 / 50 | 7 / 2 | 240 x 240 | 64 x 64 | 90 |
| Anticevic et al. (2013) | Siemens Allegra | 3.0T | Open / Rest | 210 /  3.43 x 3.43 x 4 | 1,500 / 27 | 4 / 1 | 240 x 240 | 64 x 64 | 60 |
| Baker et al. (2014) | Siemens Tim Trio | 3.0T | Open / Rest | 124 /  3 x 3 x 3 | 3,000 / 30 | 3 / 0 | 216 x 216 | - | 85 |
| Brandt et al. (2014) | Siemens Magnetom Sonata | 1.5T | Open / Task | 164 /  3 x 3 x 4 | 2,040 / 50 | 4 / 1 | 224 x 224 | 64 x 64 | 90 |
| Calhoun et al. (2012) | Siemens Allegra | 3.0T | Open / Task | - /  3.75 x 3.75 x 4 | 1,500 / 27 | 4 / 1 | 240 x 240 | 64 x 64 | 70 |
| Chai et al. (2011) | Siemens | 3.0T | Open / Rest | - /  3.5 x 3.5 x 3.5 | 2,500 / 24 | 3.5 / - | - | 64 x 64 | 82 |
| Chen et al. (2019) | GE Discovery MR750 | 3.0T | Close / Rest | 210 /  3.75 x 3.75 x 3 | 2,000 / 25 | 3 / 1 | 240 x 240 | 64 x 64 | 90 |
| Das et al. (2014) | Siemens Magnetom Trio | 3.0T | Open / Rest | 155 / - | 2,000 / 35 | 4 / 1 | - | 64 x 64 | 70 |
| Dell'Osso et al. (2015) | Philips Achieva | 3.0T | Open / Task | 276 /  1.8 x 1.8 x 5 | 2,000 / 35 | 5 / - | 230 x 230 | 96 x 96 | 90 |
| Dvorak et al. (2019) | Siemens Magnetom Trio | 3.0T | Open / Rest | 300 /  3 x 3 x 3 | 2,000 / 30 | 3 / 0.6 | - | - | 90 |
| Ellard et al. (2018) | Siemens Skyra | 3.0T | Open / Rest | - /  3 x 3 x 3 | 3,000 / 30 | 3 / - | 216 x 216 | - | 85 |
| Ellard et al. (2019) | Siemens Trio | 3.0T | Open / Task | - / - | 1,600 / 30 | 5 / 1 | - | - | 90 |
| Favre et al. (2014) | Bruker MedSpecS300 | 3.0T | Open / Rest | 144 /  3 x 3 x 3.5 | 2,500 / 30 | 3.5 / - | 216 x 216 | 72 x 72 | 77 |
|  | Philips Achieva | 3.0T | Open / Rest | 144 /  3 x 3 x 3.75 | 2,500 / 30 | 3.75 / - | 216 x 216 | 72 x 72 | 77 |
| Gong et al. (2019) | GE Discovery MR750 | 3.0T | Close / Rest | 210 /  3.75 x 3.75 x 3 | 2,000 / 25 | 3 / 1 | 240 x 240 | 64 x 64 | 90 |
| He et al. (2016) | GE Discovery MR750 | 3.0T | Open / Rest | 225 / - | 2,000 / 27 | 2.9 / - | 240 x 240 | 96 x 96 | 78 |
| He et al. (2018) | GE Discovery MR750 | 3.0T | Close / Rest | 210 / - | 2,000 / 25 | 3 / 1 | 240 x 240 | 64 x 64 | 90 |
| He et al. (2019) | GE Discovery MR750 | 3.0T | Close / Rest | 255 /  3.75 x 3.75 x 3.2 | 2,000 / 30 | 3.2 / 0 | - | 64 x 64 | 90 |
| Jimenez et al. (2019) | Siemens Tim Trio | 3.0T | - / Rest | - /  3 x 3 x 3.3 | 2,500 / 35 | 3.3 / - | 192 x 192 | 96 x 96 | 75 |
| Karcher et al. (2019) | Philips Intera Achieva | 3.0T | Open / Rest | 300 /  3 x 3 x 3 | 2,000 / 25 | 3 / 0.3 | - | 80 x 80 | 90 |
| Li G. et al. (2018) | Siemens Trio | 3.0T | Close / Rest | 212 / - | 2,000 / 30 | 3 / - | 240 x 240 | 64 x 64 | 90 |
| Li J. et al. (2018) | GE Signa HDx | 3.0T | Close / Rest | - / - | 2,000 / 30 | 3 / 0 | 240 x 240 | 64 x 64 | 90 |
| Liu et al. (2012) | Siemens Trio | 3.0T | Close / Rest | 240 / - | 2,000 / 30 | 3.5 / 0.6 | 220 x 220 | 64 x 64 | 90 |
| Liu et al. (2019) | Philips  Achieva | 3.0T | - / Rest | 250 / - | 2,000 / 30 | 4 / 0 | - | 64 x 64 | 90 |
| Lois et al. (2014) | Siemens Magnetom Trio | 3.0T | Close / Rest | 120 /  2.3 x 2.3 x 2.3 | 2,700 / 27 | 2.3 / - | 220 x 220 | 96 x 96 | 90 |
| Luo et al. (2018) | GE Discovery MR750 | 3.0T | Close / Rest | 210 /  3.75 x 3.75 x 3 | 2,000 / 25 | 3 / 1 | 240 x 240 | 64 x 64 | 90 |
| Magioncalda et al. (2015) | GE | 1.5T | Close / Rest | 150 /  3.75 x 3.75 x 4 | 2,000 / 30 | 4 / 1 | 240 x 240 | - | 90 |
| Mamah et al. (2013) | Siemens Tim Trio | 3.0T | Close / Rest | 164 /  4 x 4 x 4 | 2,500 / 27 | 4/ - | 256 x 256 | - | 90 |
| Marchand et al. (2014) | Siemens Trio | 3.0T | Open / Task | - / - | 2,080 / 30 | 3 / 0 | 220 x 220 | 64 x 64 | 75 |
| Meda et al. (2012) | Siemens Allegra | 3.0T | Open / Rest | 210 /  3.4 x 3.4 x 5 | 1,500 / 28 | 5 / - | - | - | 65 |
| Meda et al. (2014) | Siemens TrioTim | 3.0T | Open / Rest | - /  3.4 x 3.4 x 3 | 2,210 / 30 | 3 / - | - | 64 x 64 | 70 |
|  | Siemens Allegra | 3.0T | Open / Rest | - /  3.4 x 3.4 x 5 | 1,500 / 27 | 5 / - | - | 64 x 64 | 70 |
|  | Siemens TrioTim | 3.0T | Open / Rest | - /  3.4 x 3.4 x 4 | 1,570 / 22 | 4 / - | - | 64 x 64 | 60 |
|  | Philips | 3.0T | Open / Rest | - /  3.4 x 3.4 x 4 | 1,500 / 27 | 4 / - | - | 64 x 64 | 60 |
|  | GE Signa HDx | 3.0T | Open / Rest | - /  3.4 x 3.4 x 4 | 1,775 / 27 | 4 / - | - | 64 x 64 | 60 |
| Oertel-Knochel et al. (2015) | Siemens MagnetomAllegra | 3.0T | Open / Rest | 400 /  3 x 3 x 3 | 2,000 / 30 | 3 / 0.6 | - | - | 90 |
| Ongur et al. (2010) | Siemens | 3.0T | Open / Rest | 240 /  3.5 x 3.5 x 3.5 | 2,500 / 24 | 3.5 / 0 | - | - | 90 |
| Pang et al. (2018) | GE Discovery MR750 | 3.0T | Close / Rest | 255 /  3.75 x 3.75 x 3.2 | 2,000 / 30 | 3.2 / 0 | 240 x 240 | 64 x 64 | 90 |
| Pompei et al. (2011) | GE Neuro-optimised  Signa | 1.5 T | Open / Task | 104 /  3.75 x 3.75 x 7 | 3,500 / 40 | 7 / 0.7 | - | 64 x 64 | 90 |
| Radaelli et al. (2014) | Philips | 3.0T | Open / Task | 124 / - | 3,000 / 35 | 5 / - | 230 x 230 | 80 x 80 | 90 |
| Rey et al. (2016) | Siemens Magnetom Tim Trio | 3.0T | Close / Rest | 250 /  3.2 x 3.2 x 3.2 | 2,100 / 30 | 3.2 / 0.64 | 192 x 192 | 64 x 64 | 80 |
| Roberts et al. (2017) | Philips Achieva | 3.0T | Close / Rest | - / - | 2,000 / 30 | 4.5 / 0 | 250 x 250 | 136 x 136^†^ | 90 |
| Rodriguez-Cano et al. (2017) | GE Signa | 1.5T | Open / Task | 266 /  3 x 3 x 7 | 2,000 / 20 | 7 / 0.7 | - | - | 70 |
| Sheffield et al. (2017) | Siemens TrioTim | 3.0T | Open / Rest | - /  3.4 x 3.4 x 3 | 2,210 / 30 | 3 / - | - | 64 x 64 | 70 |
|  | Siemens Allegra | 3.0T | Open / Rest | - /  3.4 x 3.4 x 5 | 1,500 / 27 | 5 / - | - | 64 x 64 | 70 |
|  | Siemens TrioTim | 3.0T | Open / Rest | - /  3.4 x 3.4 x 4 | 1,570 / 22 | 4 / - | - | 64 x 64 | 60 |
|  | Philips | 3.0T | Open / Rest | - /  3.4 x 3.4 x 4 | 1,500 / 27 | 4 / - | - | 64 x 64 | 60 |
|  | GE Signa HDx | 3.0T | Open / Rest | - /  3.4 x 3.4 x 4 | 1,775 / 27 | 4 / - | - | 64 x 64 | 60 |
|  | GE Signa HDx | 3.0T | Open / Rest | - /  3.4 x 3.4 x 5 | 3,000 / 27 | 5 / - | - | 64 x 64 | 60 |
| Skatun et al. (2018) | GE Signa HDxt | 3.0T | Open / Rest | 203 /  4 x 4 x 3 | 2,638 / 30 | 3 / - | - | 64 x 64 | 90 |
| Thomas et al. (2019) | Siemens Tim Trio | 3.0T | Open / Rest | - /  3 x 3 x 3 | 2,000 / 25 | 3 / - | 192 x 192 | - | 90 |
| Townsend et al. (2013) | Siemens Allegra | 3.0T | Open / Task | - /  3.75 x 3.75 x 3 | 2,500 / 35 | 3 / 1 | 240 x 240 | 64 x 64 | 90 |
| Wang J et al. (2018) | GE Discovery MR750 | 3.0T | Close / Rest | 210 / - | 2,000 / 25 | 3 / 1 | 240 x 240 | 64 x 64 | 90 |
| Wang J et al. (2019a) | GE Discovery MR750 | 3.0T | Close / Rest | 210 / - | 2,000 / 25 | 3 / 1 | 240 x 240 | 64 x 64 | 90 |
| Wang J et al. (2019b) | GE Discovery MR750 | 3.0T | Close / Rest | 210 / - | 2,000 / 25 | 3 / 1 | 240 x 240 | 64 x 64 | 90 |
| Wang X et al. (2019) | Siemens Magnetom  Trio | 3.0T | Close / Rest | 212 / - | 2,000 / 30 | 3 / 1 | 240 x 240 | 64 x 64 | 90 |
| Wang Y et al. (2018) | GE Discovery MR750 | 3.0T | Close / Rest | 210 /  3.75 x 3.75 x 3 | 2,000 / 25 | 3 / 1 | 240 x 240 | 64 x 64 | 90 |
| Yip et al. (2014) | Siemens Trio | 3.0T | - / Rest | - /  3 x 3 x 3.5 | 2,000 / 28 | 3.5 / - | 192 x 192 | - | 89 |

Device information and acquisition parameters of the functional magnetic resonance imaging data as provided by the articles currently reviewed. Studies with missing information of the above parameters are noted in dashed lines.

Abbreviations: fMRI, functional magnetic resonance imaging; FA, flip angle; FOV, field of view; TR, repetition time; TE, echo time.

^†^Parameter represented in Fourier space.

**Supplementary Table 2. Coordinates of regions with significantly altered functional activity**

| **Brain Region** | **MNI Coordinate** | | | **Cluster Size**  (voxel unless started otherwise) | |
| --- | --- | --- | --- | --- | --- |
|  | **X** | **Y** | **Z** |  |  |
| Anterior cingulate cortex L | -2 | 3 | 50 | 3544 |  |
|  | -6 | 33 | 33 | 81 |  |
|  | -18 | 46 | 10 | - |  |
| Anterior cingulate cortex R | 8 | 20 | 28 | - |  |
| Amygdala L | -21 | -5 | -12 | - |  |
| Amygdala R | 24 | -5 | -10 | - |  |
| Cerbellum vermis I | -9 | -78 | -24 | 66 |  |
|  | -24 | -78 | -21 | 3726 |  |
| Cerebellum vermis II | -6 | -78 | -36 | 1944 |  |
| Cerebellum vermis VI | -33 | -54 | -30 | 1944 |  |
|  | 24 | -54 | -21 | 3780 |  |
| Cerebellum vermis VIIIa | 33 | -42 | -48 | 3861 |  |
| Cerebellum vermis IV, V | 6 | -60 | -12 | 111 |  |
| Cerebellum vermis IX.L | -6 | -48 | -45 | 1404 |  |
|  | -9 | -54 | -51 | 63 |  |
|  | -21 | -30 | -48 | 2133 |  |
| Cuneus L | -12 | -69 | 27 | 58 |  |
| Cuneus R | 1 | -63 | 45 | 45 |  |
| Caudate L | -16 | 22 | 10 | 132 |  |
| Caudate R | 14 | 14 | 14 | 274 |  |
| Dorsal anterior cingulate cortex L | -12 | 52 | 2 | 83 |  |
| Dorsal anterior cingulate cortex R | 12 | 28 | 20 | 548 |  |
| Dorsolateral prefrontal cortex L | -32 | 2 | 50 | 158 |  |
|  | -33 | 6 | 48 | 2160 |  |
|  | -44 | 46 | 13 | - |  |
|  | -48 | 27 | 18 | 810 |  |
|  | -52 | 42 | 10 | 49 |  |
|  | -52 | 10 | 18 | - |  |
| Dorsolateral prefrontal cortex R | 30 | 21 | 48 | 69 |  |
|  | 34 | 43 | 30 | - |  |
|  | 42 | 41 | 14 | 67 |  |
|  | 45 | 33 | 36 | 29 |  |
|  | 50 | 36 | 18 | 55 |  |
| Dorsomedial prefrontal cortex L | -14 | 44 | 26 | 814 |  |
| Frontal pole L | -9 | 57 | 42 | 34 |  |
|  | -36 | 18 | 51 | 1012 |  |
| Frontal pole R | 12 | 36 | 60 | 102 |  |
|  | 18 | 54 | 33 | 61 |  |
|  | 18 | 57 | 36 | 69 |  |
| Hippocampus L | -15 | -36 | 9 | 42 |  |
|  | -24 | -30 | -6 | 1512 |  |
|  | -27 | -42 | -3 | 1869 |  |
|  | -34 | -38 | -4 | 56 |  |
| Hippocampus R | 27 | -27 | -6 | 1917 |  |
|  | 32 | 26 | 34 | 1416 |  |
|  | 34 | -22 | -14 | 217 |  |
|  | 36 | -18 | -18 | 1056 |  |
| Insula L | -34 | 8 | -2 | - |  |
|  | -38 | 12 | 4 | 51 |  |
| Insula R | 34 | -15 | 6 | - |  |
| Inferior frontal gyrus L | -25 | 32 | -9 | - |  |
| Inferior parietal lobe L | -40 | -36 | 38 | 173 |  |
|  | -48 | -48 | 48 | - |  |
| Inferior parietal lobe R | 51 | -54 | 48 | 29 |  |
|  | 56 | -44 | 46 | 271 |  |
|  | 57 | -36 | 48 | 1404 |  |
| Inferior temporal gyrus L | -54 | -15 | -30 | 594 |  |
|  | -55 | -10 | -13 | 85 |  |
| Inferior temporal gyrus R | 44 | -4 | -34 | 384 |  |
|  | 45 | 6 | -45 | 702^†^ |  |
| Medial frontal gyrus L | -24 | 18 | 42 | 624 |  |
|  | -27 | 27 | 33 | 41 |  |
|  | -28 | 4 | 60 | - |  |
|  | -36 | 15 | 51 | 107 |  |
|  | -36 | 15 | 51 | 38 |  |
| Medial frontal gyrus R | 4 | 58 | 10 | - |  |
|  | 32 | 0 | 60 | - |  |
|  | 34 | 42 | 10 | - |  |
|  | 42 | 18 | 42 | 102 |  |
| Middle occipital gyrus L | -18 | -67 | 17 |  |  |
| Middle occipital gyrus R | 27 | 96 | 15 | 28 |  |
| Medial prefrontal cortex L | -1 | 49 | -2 | - |  |
|  | -2 | 8 | 46 | - |  |
|  | -24 | 42 | 45 | 1161 |  |
| Medial prefrontal cortex | 0 | 50 | 1 |  |  |
| Medial prefrontal cortex R | 1 | 41 | -3 | - |  |
|  | 4 | 14 | 48 | - |  |
|  | 5 | 34 | 44 | 106 |  |
|  | 18 | 63 | 12 | 10,989^†^ |  |
| Middle temporal gyrus L | -40 | -61 | 27 | 2071 |  |
|  | -69 | -39 | -9 | 1242 |  |
| Middle temporal gyrus R | 44 | -59 | 23 | 1695 |  |
|  | 57 | 6 | 27 | 37 |  |
| Perigenual anterior cingulate cotex | 0 | 45 | 0 | - |  |
|  | 9 | 39 | 9 | 397 |  |
| Posterior cingulate cortex L | -5 | -50 | 36 | - |  |
| Posterior cingulate cortex | 0 | -56 | 20 | - |  |
| Posterior cingulate cortex R | 6 | -57 | 6 | 3024 |  |
|  | 21 | -67 | 14 | - |  |
| Paracentral lobule L | -3 | -24 | 51 | 3186 |  |
| Precuneus L | -6 | -66 | 45 | 165 |  |
| Precuneus R | 3 | -57 | 30 | 3051^†^ |  |
|  | 6 | -75 | 39 | 108 |  |
|  | 9 | -51 | 72 | 345 |  |
|  | 9 | -51 | 57 | 32 |  |
| Putamen L | -20 | 6 | -14 | - |  |
|  | -24 | 6 | -6 | - |  |
| Putamen R | 24 | 9 | 9 | 1593 |  |
| Subgenual anterior cingulate cortex L | -4 | 21 | -8 | - |  |
|  | -4 | 23 | 35 | 848 |  |
|  | -5 | 25 | -10 | - |  |
| Subgenual anterior cingulate cortex R | 5 | 25 | 10 | - |  |
| Superior temporal gyrus L | -25 | 8 | 58 | - |  |
| Superior temporal gyrus R | 66 | -3 | 6 | 1080 |  |
| Thalamus L | -3 | -9 | -6 | 298 |  |
|  | -3 | -15 | 6 | 337 |  |
| Ventrolateral prefrontal cortex L | -34 | 27 | -8 | - |  |
|  | -40 | 23 | 14 | 371 |  |
|  | -48 | 2 | 5 | - |  |
| Ventrolateral prefrontal cortex R | 27 | 35 | -1 |  |  |
|  | 36 | 31 | -8 | - |  |
|  | 40 | 20 | -4 | 104 |  |
|  | 40 | 26 | -12 | - |  |
|  | 54 | 18 | 0 | 37 |  |
| Ventromedial prefrontal cortex L | -12 | 52 | -2 | 34 |  |
|  | -15 | 57 | 12 | 324 |  |
| Ventromedial prefrontal cortex R | 16 | 52 | -2 | 100 |  |

Brain coordinates of the seed regions that were considered to undergo significant alteration in functional activity or connectivity as provided by a number of the articles currently reviewed. Studies that provided brain coordinates using the Talairach coordinates were translated to MNI coordinates accordingly. Cluster size of the seed regions were presumed to be in the unit of voxel unless stated otherwise.

Abbreviations: L, left; MNI, Montreal Neurological Institute; R, right.

^†^Cluster size in the unit of cubic millimeter (mm^3^).
